# Anaplastic oligodendrogliomas with 1p19q codeletion have a proneural gene expression profile

**DOI:** 10.1186/1476-4598-7-41

**Published:** 2008-05-20

**Authors:** François Ducray, Ahmed Idbaih, Aurélien de Reyniès, Ivan Bièche, Joëlle Thillet, Karima Mokhtari, Séverine Lair, Yannick Marie, Sophie Paris, Michel Vidaud, Khê Hoang-Xuan, Olivier Delattre, Jean-Yves Delattre, Marc Sanson

**Affiliations:** 1 Unité INSERM U711, Université Paris VI, 47-83 Boulevard de l'Hôpital, 75013 Paris, France; 2 Programme Cartes d'Identité des Tumeurs (CIT), Ligue Nationale Contre le Cancer, Service de Bioinformatique, 14 rue Corvisart, 75014 Paris, France; 3 Unité INSERM U745, Faculté des Sciences Pharmaceutiques et Biologiques, Université René Descartes – Paris V, 4 avenue de l'Observatoire 75270 Paris, France; 4 Unité INSERM U830, Unité de Génétique Somatique et Service de Bio-Informatique, Institut Curie, 26 rue d'Ulm, 75006 Paris, France

## Abstract

**Background:**

In high grade gliomas, 1p19q codeletion and *EGFR *amplification are mutually exclusive and predictive of dramatically different outcomes. We performed a microarray gene expression study of four high grade gliomas with 1p19q codeletion and nine with *EGFR *amplification, identified by CGH-array.

**Results:**

The two groups of gliomas exhibited very different gene expression profiles and were consistently distinguished by unsupervised clustering analysis. One of the most striking differences was the expression of normal brain genes by oligodendrogliomas with 1p19q codeletion. These gliomas harbored a gene expression profile that partially resembled the gene expression of normal brain samples, whereas gliomas with *EGFR *amplification expressed many genes in common with glioblastoma cancer stem cells. The differences between the two types of gliomas and the expression of neuronal genes in gliomas with 1p19q codeletion were both validated in an independent series of 16 gliomas using real-time RT-PCR with a set of 22 genes differentiating the two groups of gliomas (*AKR1C3*, *ATOH8*, *BMP2*, *C20orf42*, *CCNB1*, *CDK2*, *CHI3L1*, *CTTNBP2*, *DCX, EGFR, GALNT13, GBP1, IGFBP2, IQGAP1, L1CAM, NCAM1, NOG, OLIG2, PDPN, PLAT, POSTN, RNF135*). Immunohistochemical study of the most differentially expressed neuronal gene, alpha-internexin, clearly differentiated the two groups of gliomas, with 1p19q codeletion gliomas showing specific staining in tumor cells.

**Conclusion:**

These findings provide evidence for neuronal differentiation in oligodendrogliomas with 1p19q codeletion and support the hypothesis that the cell of origin for gliomas with 1p19q codeletion could be a bi-potential progenitor cell, able to give rise to both neurons and oligodendrocytes.

## Background

The 1p19q codeletion and *EGFR *amplification are mutually exclusive and related to dramatically different outcomes in high grade gliomas. The 1p19q codeletion is strongly associated with an oligodendroglial phenotype and favorable prognosis [1]. It has recently been shown to be mediated by a specific t(1;19)(q10;p10) translocation [2]. To date the efforts performed to identify the genes specifically involved in the breakpoint have failed, mostly because both 1p and 19q centromeric regions contain highly repeated sequences. As a consequence the molecular mechanisms underlying the particular phenotype and the favorable outcome of this subset of gliomas remain completely unknown. Reliable detection of 1p19q codeletion requires an appropriate technique, such as CGH-array. Indeed, the most widely used LOH studies may not distinguish this signature from partial distal 1p and 19q deletion or gain, which have radically different prognostic implications [1]. On the other hand, *EGFR *amplification is tightly associated with chromosome 10 loss and gain of chromosome 7, representing another characteristic genomic signature [3]. *EGFR *amplification is more frequent in glioblastomas, but it is also found in a subset of anaplastic oligodendrogliomas and, in this setting, is predictive of extremely poor prognosis [4]. Recently, malignant gliomas have been separated into three expression profiles with distinct outcomes and histological correlations: 1) the proneural profile with a better prognosis, mostly corresponding to anaplastic gliomas (oligodendrogliomas and astrocytomas); 2) the proliferative and 3) mesenchymal profiles, corresponding mainly to glioblastomas [5]. However, correlation with 1p19q codeletion is still missing. Based on a set of gliomas analyzed by CGH-array [3], we selected tumors displaying one of these two characteristics and mutually exclusive patterns -1p19q codeletion or *EGFR *amplification- and compared their gene expression profiles.

## Methods

### Samples

The microarray study was done on 13 gliomas selected from the Salpêtrière database, based on the following criteria: 1) CGH-array profile showing either whole 1p19q codeletion or *EGFR *amplification, 2) high quality RNA availability. The samples were provided as snap-frozen sections of areas immediately adjacent to the region used for the histopathological diagnosis according to the World Health Organization Classification (WHO 2000). This set included 4 grade III oligodendrogliomas with complete 1p19q codeletion and 9 gliomas with *EGFR *amplification (5 glioblastomas (GBM), 3 grade III oligodendrogliomas, 1 grade III oligoastrocytoma (OAIII)). Genomic characterization was performed using CGH array as previously described [1]. Among the 9 tumors with *EGFR *amplification, 8 out of 9 had chromosome 10q loss and chromosome 7 gain; 4 had a gain of chromosome 1p, and 4 had a partial loss of chromosome 1p. Among the 4 tumors with complete 1p19q codeletion none had *EGFR *amplification, 10q loss or chromosome 7 gain, and 2 had complete chromosome 4 loss. In order to compare the gene expression profile of the gliomas with normal brain, we used the gene expression data of 5 samples of corpus callosum (GSM175855, GSM175856, GSM175857, GSM175858, GSM176050) and 5 samples of cortex (GSM176049, GSM176344, GSM176345, GSM176346, GSM176347), available in the Gene Expression Omnibus repository (GSE7307) [6]. To compare the gene expression profile with glioblastomas cancer stem cells (CSC), we used the data of Beier et al. (GSE7181) [7]. All raw and normalized data files for the microarray analysis have been deposited [8] at the European Bioinformatics Institute (Hinxton, UK), and are publicy available under accession number E-MEXP-1507.

### RNA extraction and hybridization

Approximately 50 mg of tissue from each tumor were used for total RNA extraction using the RNeasy Lipid Tissue mini kit (Qiagen, CA), according to the manufacturer instructions. RNA quality was verified with the Bioanalyser System (Agilent Technologies, Paolo Alto, CA), using the RNA Nano Chips. One and half micrograms of RNA were processed for hybridization on the Genechip Human Genome U133 Plus 2.0 Expression array (Affymetrix, CA), which contains over 54.000 probe sets analyzing the expression level of over 47,000 transcripts and variants, including 38.500 well-characterized human genes. The processing was done according to the recommendations of the manufacturer.

### Data analysis

Except as indicated, all transcriptome analysis was carried out using either R-system software (version 2.4.1) packages including those of Bioconductor [9]or original R code (A. de R.). Normalization was performed using the RMA method [10]. Clustering analysis was performed as previously reported [11]. Class comparison using a univariate t-test was performed using BRB Array Tools developed by Dr. Richard Simon and Amy Peng Lam [12]. A p-value < 0.001 was used to define differentially expressed genes. Gene set enrichment analysis was performed using GSEA v2.0 software [13] as described by Subramanian et al. [14]. For enrichment analysis in specific gene ontology terms (GO terms), we used a hypergeometric test to measure the association between a gene (probe set) list and a GO term. To this end, we mapped both the gene list and GO term related proteins to non-redundant Entrez Gene identifiers. The mapping of a probe set list to Entrez Gene ids was done using the annotation file HG-U133_Plus_2.annot.csv [15]. For each GO term, we obtained the list of non-redundant related protein identifiers – either directly associated with the GO term or with one of its descendants – and mapped it to a non-redundant list of Entrez Gene ids. GO terms and their relationships (parent/child) were downloaded from The Gene Ontology site [16]. The list of proteins associated with GO terms (table gene_association.goa_human) was downloaded from [17]. We designated a threshold significance level for the hypergeometric test of p < 0.01 and the criteria that a GO term was represented by at least 2 Entrez Gene identifiers. Enrichment analysis of genes located on specific chromosomes was performed using DAVID tools [18].

### Real-time RT-PCR

Quantitative RT-PCR was performed as previously described [19]. *TBP *(Genbank accession no. NM_003194), which encodes the TATA box-binding protein (a component of the DNA-binding protein complex transcription factor II D), was selected as an endogenous control because the levels of its transcript did not change across various normal tissues and tumor samples. The expression of the following 22 genes was quantified: *AKR1C3 *(upper primer (UP) 5'-CGT ATT TCA ACC GGA GTA AAT TGC TA-3', lower primer (LP) 5'-GTT CGG GTC CAC CCA TCG T-3'); *ATOH8 *(UP 5'-CAC ACC ATC AGC GCA GCC TT-3', LP 5'-GAT GGC CAG TTT GGA CAG CTT CT-3'); *BMP2 *(UP 5'-CGC AGC TTC CAC CAT GAA GAA TC-3', LP 5'-GAA TCT CCG GGT TGT TTT CCC ACT-3'); *C20orf42 *(UP 5'-AAG GAA CTT GAA CAA GGA GAA CCA CT-3', LP 5'-GGC ACA ACT TCG CAG CCT CTA-3'); *CCNB1 *(UP 5'-TGG ATA ATG GTG AAT GGA CAC CAA-3', LP 5'-GCC AGG TGC TGC ATA ACT GGA-3'); *CDK2 *(UP 5'-GGA CGG AGC TTG TTA TCG CAA AT-3', LP 5'-CCT TGG CCG AAA TCC GCT T-3'); *CHI3L1 *(UP 5'-GAC CAC AGG CCA TCA CAG TCC-3', LP 5'-TGT ACC CCA CAG CAT AGT CAG TGT T-3'); *CTTNBP2 *(UP 5'-CCC TCT CCA TCC TTG AAG CAG T-3', LP 5'-GAA GCT TCT CCA TTT CCA GCT TCT-3'); *DCX *(UP 5'-AGC CAA GAG CCC TGG TCC TAT-3', LP 5'-TGG AGG TTC CGT TTG CTG AGT-3');*EGFR *(UP 5'-GGA GAA CTG CCA GAA ACT GAC C-3', LP 5'-GCC TGC AGC ACA CTG GTT G-3');*GALNT13 *(UP 5'-GTG GCC TAT TTT CTA TTG ACA GAA ACT-3', LP 5'-CCT CCA CAT TGC CAA ATC CTA A-3');*GBP1 *(UP 5'-CCT CGC TCT TAA ACT TCA GGA ACA-3', LP 5'-CCT TTC GTC GTC TCA TTT TCG T-3');*IGFBP2 *(UP 5'-GGC CCT CTG GAG CAC CTC TAC T-3', LP 5'-CCG TTC AGA GAC ATC TTG CAC TGT-3');*IQGAP1 *(UP 5'-AGA ACA GAC CAG ATA CAA GGC GA-3', LP 5'-CTT AGG CAA TCC AAT CTC ATC CA-3');*L1CAM *(UP 5'-CTG GTT GTC TTC CCC ACA GAT GA-3', LP 5'-TCG TCC AGC GGA ACT GCA CT-3');*NCAM1 *(UP 5'-CCA CAG CCA TCC CAG CCA A-3', LP 5'-GAC GAT GAG GAT GCC CAC GAT-3');*NOG *(UP 5'-AAG AAG CAG CGC CTA AGC AAG A-3', LP 5'-GTC GTT CCA CGC GTA CAG CA-3');*OLIG2 *(UP 5'-CGC CAG AGC CCG ATG ACC TT-3', LP 5'-GAC ACG GTG CCC CCA GTG AA-3');*PDPN *(UP 5'-GTG ACT CCA GGA ACC AGC GAA-3', LP 5'-TGA CAC TTG TTG CCA CCA GAG TT-3');*PLAT *(UP 5'-AGC AGG CCC TGT ACT TCT CAG ATT-3', LP 5'-ACG TGG CCC TGG TAT CTA TTT CA-3');*POSTN *(UP 5'-GTC CTA ATT CCT GAT TCT GCC AAA-3', LP 5'-GGG CCA CAA GAT CCG TGA A-3');*RNF135 *(UP 5'-TGC CTG ACC AGA GCC ACC C-3', LP 5'-GAT GGA TGG CCC ACT GAG CA-3'); *TBP *(UP 5'-TGC ACA GGA GCC AAG AGT GAA-3', LP 5'-CAC ATC ACA GCT CCC CAC CA-3'). RT-PCR validation was done in an independent set of 16 gliomas (8 grade III oligodendrogliomas with complete 1p19q codeletion and 8 glioblastomas with *EGFR *amplification) and on 3 samples of normal brain (temporal lobe obtained during surgery for epilepsy).

### Immunohistochemistry

Immunohistochemical analyses were carried out on paraffin sections using antibodies directed against internexin neuronal intermediate filament protein alpha (INA) [20]. Immunostaining was studied in five glioblastomas with *EGFR *amplification and five anaplastic oligodendrogliomas with complete 1p19q codeletion.

## Results

### Transcriptomic differences partly reflect the underlying genomic alterations

**Figure 1:**
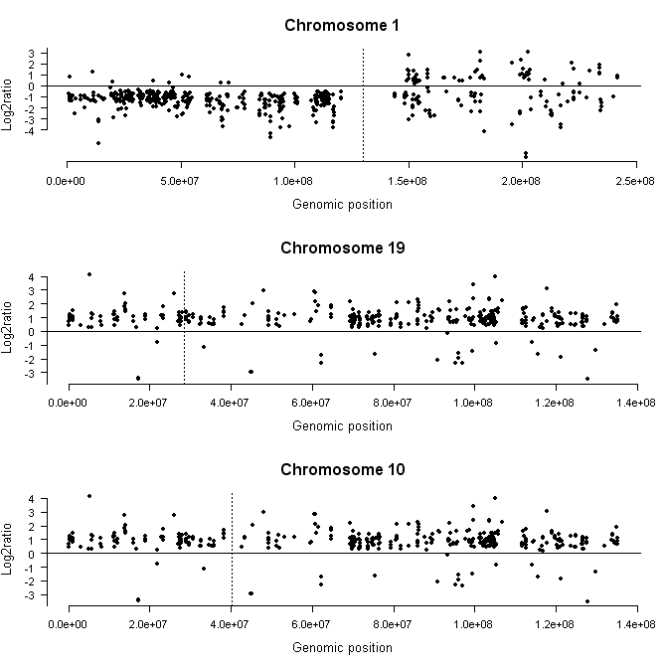
**Genomic localization of the differentially expressed probe sets (p < 0.001) localized on chromosome 1, 19 and 10**. Each probe set is represented by a dot. Probe sets are ordered along the x axis according to their genomic position (only probe sets with unambiguous genomic mapping on UCSC were used). For each chromosome, the telomere of the short arm is on the left, and the telomere of the long arm is on the right. The dashed vertical line represents the position of the centromere. The y axis corresponds to the log2 ratio of the geometric mean in the gliomas with complete 1p19q loss versus the gliomas with *EGFR *amplification. Almost all probe sets localized on 1p and 19q were underexpressed in gliomas with 1p19q codeletion, whereas most of the probe sets localized on chromosome 10 were overexpressed.

To study the relationship between the differences in gene expression profile and the underlying genomic alterations, we looked at the genomic localization of the differentially expressed genes. Class comparison using a univariate t-test demonstrated that 4458 probe sets were differentially expressed between the two groups of gliomas with a p-value < 0.001 and a maximum false discovery rate of 1.2% (see Additional file 1). The set of overexpressed genes in gliomas with *EGFR *amplification was significantly enriched in genes located on chromosome 1 (305 genes, p < 10^-4^), chromosome 19 (151 genes, p < 10^-4^), chromosome 7 (109 genes, p < 10^-4^) and chromosome 4 (85 genes, p < 10^-4^). In contrast, the set of overexpressed genes in gliomas with 1p19q codeletion was significantly enriched in genes located on chromosome 10 (216 genes, p < 10^-4^). Thus, gene expression and copy number variation dynamics were tightly correlated in both tumor groups. This was even more obvious when we plotted the differentially expressed probe sets localized on chromosome 1, 19 and 10 according to their genomic localization and the log2 ratio of their geometric mean in both tumor groups (Figure 1). Indeed, almost all differentially expressed genes localized on 1p and 19q were underexpressed in gliomas with 1p19q codeletion, whereas differentially expressed genes localized to 1q and 19p were equally distributed (Figure 1).

### Unsupervised analysis consistently distinguishes the two groups of gliomas

**Figure 2:**
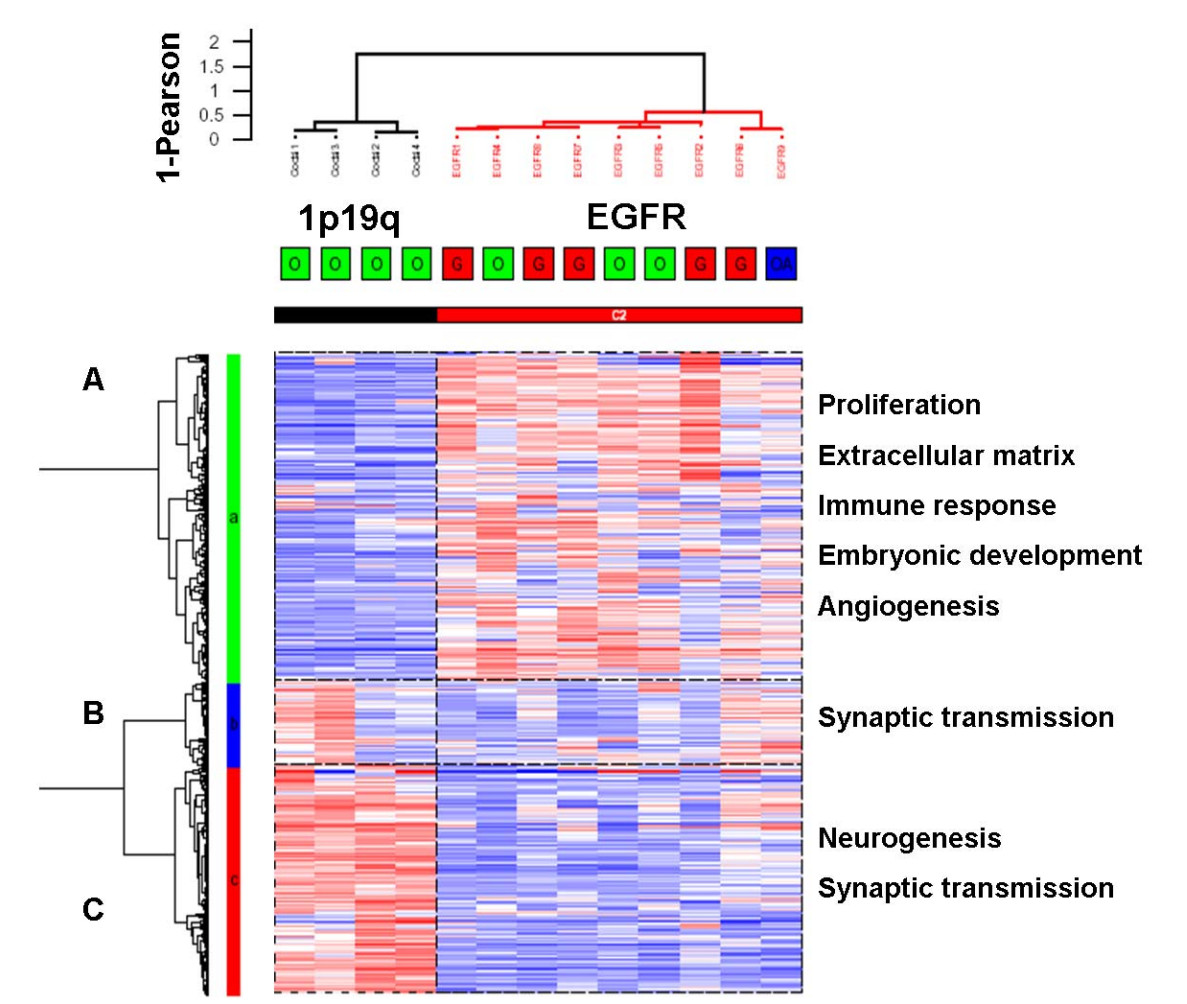
**Unsupervised clustering of 4 oligodendrogliomas with 1p19q codeletion and 9 gliomas with *EGFR *amplification**. Unsupervised hierarchical clustering was performed using the 1366 probe sets whose expression varied the most across the 13 samples (probe sets with a robust coefficient of variation superior to the 97.5^th ^percentile). Samples and genes were clustered using Ward's linkage and 1-Pearson correlation coefficient. For each probe set, data were median-centered (white), with the lowest and highest intensity values in blue and red, respectively. 1p19q = 1p19q codeletion, *EGFR *= *EGFR *amplification. The gliomas were classified into 2 groups according to their genomic profile. Gliomas with *EGFR *amplification were classified into one cluster irrespective of their histology (red = glioblastoma, green = grade III oligodendroglioma, blue = grade III oligoastrocytoma). Gene cluster A was enriched in genes involved in proliferation, extracellular matrix, immune response, embryonic development and angiogenesis. Gene cluster B was enriched in genes involved in synaptic transmission. Gene cluster C was enriched in genes involved in neurogenesis and synaptic transmission.

In order to study the differences in gene expression profile, we performed unsupervised hierarchical clustering analysis of the 13 glioma samples. This analysis was done using the 1366 probe sets whose expression varied the most across samples (this corresponds to the probe sets with a robust coefficient of variation (rCV) superior to the 97.5^th ^rCV percentile). As shown in Figure 2, gliomas with 1p19q codeletion and gliomas with *EGFR *amplification segregated into two distinct groups. This clustering was extremely robust and was conserved across different gene lists and clustering methods. The genes were classified into three clusters: one cluster of genes overexpressed in gliomas with *EGFR *amplification (gene cluster A, 698 genes), one cluster of genes overexpressed in oligodendrogliomas with 1p19q codeletion (gene cluster C, 488 genes) and a smaller cluster of genes expressed by some samples of both groups (gene cluster B, 180 genes). Enrichment analysis was performed on these three gene clusters. First, chromosome enrichment analysis demonstrated that these gene clusters did not simply reflect the underlying genomic alterations. Indeed, gene cluster A was enriched in genes located on chromosome 4 (53 genes, p < 10^-4^) and on chromosome 7 (43 genes, p < 10^-2^), but not in genes located on chromosome 1 or chromosome 19. Neither gene cluster C nor gene cluster B was enriched in genes located on chromosome 10. In contrast, the three gene clusters were enriched in genes with specific ontologic classes. Gene cluster A was most significantly enriched in genes involved in: immune response (55 genes, p < 10^-4^), extracellular matrix (37 genes, p < 10^-4^), proliferation (45 genes, p < 10^-4^), blood vessel development (17 genes, p < 10^-4^) and embryonic development (14 genes, p < 10^-4^). This cluster contained several genes well-known to be overexpressed in glioblastomas (*IGFBP2, CHI3L1*) and markers of glioblastoma cancer stem cells as well (*CD133, IQGAP1*). In contrast, gene cluster C was significantly enriched in genes with different specific ontologic classes: nervous system development (42 genes, p < 10^-4^), synaptic transmission (26 genes, p < 10^-4^) and neurogenesis (13 genes, p < 10^-4^). Actually, most of the genes in gene cluster C were either related to neuronal function or neuronal development or known to be highly expressed in normal brain. Gene cluster B was also enriched in genes involved in synaptic transmission (17 genes, p < 10^-4^) and nervous system development (16 genes, p < 10^-4^). However, two ontologic classes specifically found in this cluster (neurofilament (3 genes, p < 10^-4^) and axon ensheathment (2 genes, p < 10^-2^)) suggested that this cluster contained genes expressed in more differentiated neural cells than gene cluster C.

### Oligodendrogliomas with 1p19q codeletion express specific subsets of neuronal genes

**Figure 3:**
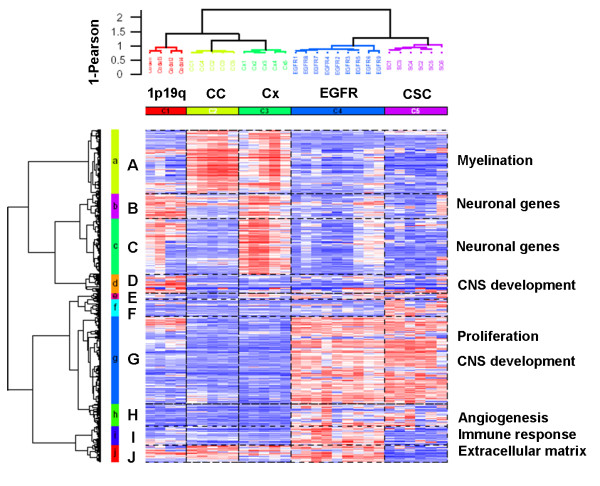
**Unsupervised clustering of 4 gliomas with 1p19q codeletion, 9 gliomas with *EGFR *amplification, 6 glioblastoma cancer stem cells cell lines and 10 normal brain tissue samples**. Unsupervised hierarchical clustering was performed using the 1366 probe sets whose expression varied the most across the 29 samples (probe sets with a robust coefficient of variation superior to the 97.5^th ^percentile). Samples and genes were clustered using Ward's linkage and 1-Pearson correlation coefficient. For each probe set, data were median-centered (white), with the lowest and highest intensity values in blue and red, respectively. 1p19q = 1p19q codeletion, *EGFR *= *EGFR *amplification, CC = corpus callosum, Cx = cortex, CSC = cancer stem cells. The 29 gliomas were classified into 2 groups and 5 subgroups. Gliomas with *EGFR *amplification were classified with the cancer stem cell lines. Gliomas with 1p19q codeletion were classified with the normal brain samples, however their gene expression pattern was clearly different from the gene expression pattern of the white matter (corpus callosum) and grey matter (cortex) samples.

In order to better characterize the expression of neuronal genes in gliomas with complete 1p19q codeletion, we performed a new hierarchical clustering analysis with samples of normal brain (GSE7307), including grey matter (cortex) and white matter (corpus callosum). As glioblastomas expressed genes of neural cancer stem cells, we also included samples of glioblastoma cancer stem cells (GSE7181) [7] in this analysis. Still using the most differentially expressed 1366 probe sets, the samples clustered into two major groups: one containing 1p19q codeleted gliomas, normal white matter and normal grey matter, and the other containing *EGFR *amplified gliomas and cancer stem cells (Figure 3). Again this clustering was found to be extremely robust and was conserved across different gene lists and clustering methods. Gene ontology enrichment analysis was performed on the main gene clusters (A to J). Despite the clustering of 1p19q codeleted gliomas with normal brain, they showed substantial differences. First, the genes characteristics of the corpus callosum (gene cluster A), enriched in myelination genes (5 genes, p < 10^-4^), were not overexpressed in gliomas with 1p19q codeletion. Second, gliomas with 1p19q only overexpressed one subset of the neuronal genes characteristic of the cortex samples (gene clusters B and C). For example, among the neurofilament genes (*INA, NEFH, NEFM, NEFL*) present in cluster C, alphainternexin (*INA*) was the only one overexpressed in both gliomas with 1p19q codeletion and cortex samples in comparison to gliomas with *EGFR *amplification. Third, 1p19q codeleted gliomas were characterized by one specific cluster (gene cluster D) enriched in genes involved in CNS development (6 genes, p < 10^-4^), which also contained genes known to be specifically expressed in neuronal cells in the physiological state, e.g. *DCX *and *GALNT1 *[21,22]. Thus, gliomas with 1p19q codeletion had a specific gene expression pattern of neuronal genes different from the samples of normal brain. Gliomas with *EGFR *amplification and glioblastoma cancer stem cells were both characterized by a large gene cluster (G) most significantly enriched in genes involved in proliferation (34 genes, p < 10^-4^) and in CNS development (16 genes, p < 10^-4^). Gliomas with *EGFR *amplification segregated from the cancer stem cells by one main gene cluster (I) enriched in genes involved in immune response (11 genes, p < 10^-4^), extracellular matrix (7 genes, p < 10^-4^) and angiogenesis (4 genes, p < 10^-4^).

### Most characteristic genes associated with oligodendrogliomas with 1p19q codeletion

To find the genes most specifically associated with oligodendrogliomas with 1p19q codeletion, we selected the probe sets that were consistently (> 2-fold) and significantly (t test p < 0.001) overexpressed in these gliomas when they were independently compared to each of the 4 other samples groups (i.e., gliomas with *EGFR *amplification, cortex samples, corpus callosum samples and glioblastoma cancer stem cells). Eighty-six probe sets corresponding to 39 well-annotated genes met these criteria (Table 1 and see additional file 1). Several genes on this list are known to be highly expressed in normal brain (*CSMD3, C20orf42, CTTNBP2*), and one is known to be specifically expressed by neuronal cells (*GALNT13*) [22]. Two transcription factors that play a role in CNS development were also specifically overexpressed (*ATOH8, NFIB*). *ATOH8 *is a basic-helix-loop-helix transcription factor, whose homolog in mouse has been demonstrated to regulate neuronal versus glial fate [23]. *NFIB *plays a role in brain development, and *Nfib *deficient mice exhibit callosal agenesis [24]. Finally, an intriguing feature was the specific overexpression of both *BMP2*, which promotes astroglial differentiation, and its antagonist *NOG*, which has been shown to promote both neuronal and oligodendroglial differentiation [25-27].

**Table 1:** Most characteristic genes associated with oligodendrogliomas with 1p19q codeletion

**Probe set**	**Title**	**Gene Symbol**	**Gene ontology (biological process)**	**High expression in:**	**FD/EGFR**	**FD/Cx**	**FD/CC**	**FD/Stem cells**
206785_s_at	Killer cell lectin-like receptor subfamily C, member 1///member 2	***KLRC1//KLRC2***	Cellular defense response	Natural killer cells	**104.2**	92.1	61.4	66.7
243779_at, 236536_at	UDP-N-acetyl-alpha-D-galactosamine:polypeptide N-acetylgalactosaminyltransferase 13 (GalNAc-T13)	***GALNT13***	Protein amino acid O-linked glycosylation	Specifically expressed in neuronal cells	**31.8**	18.2	17.1	15.0
1558706_a_at, 228890_at	Atonal homolog 8 (Drosophila)	***ATOH8***	Regulation of transcription	---	**26.1**	30.8	21.4	27.1
240228_at	CUB and Sushi multiple domains 3	***CSMD3***	Integral to membrane	Brain	**22.7**	11.8	13.9	15.1
207723_s_at	Killer cell lectin-like receptor subfamily C, member 3	***KLRC3***	Cellular defense response	Natural killer cells	**17.9**	27.3	13.1	11.3
230826_at	Monocyte to macrophage differentiation-associated 2	***MMD2***	Cytolysis	---	**17.2**	7.2	9.1	26.2
60474_at, 218796_at	Chromosome 20 open reading frame 42	***C20orf42***	Cell adhesion	Brain (among others)	**16.2**	37.4	29.1	19.6
231798_at	Noggin	***NOG***	Nervous system development	---	**11.2**	9.2	9.5	13.6
1556599_s_at	Cyclic AMP-regulated phosphoprotein, 21 kD	***ARPP-21***	---	---	**11.1**	14.0	16.8	13.9
227845_s_at	Src homology 2 domain containing transforming protein D	***SHD***	Intracellular signaling cascade	---	**10.3**	10.6	16.5	7.6
205289_at, 205290_s_at	Bone morphogenetic protein 2	***BMP2***	Positive regulation of astrocyte differentiation	Brain (among others)	**10.1**	30.7	25.9	12.7
205330_at	Meningioma (disrupted in balanced translocation) 1	***MN1***	Negative regulation of progression through cell cycle	Ubiquitously expressed	**8.4**	5.8	9.5	15.9
219668_at	Ganglioside-induced differentiation-associated protein 1-like 1	***GDAP1L1***	---	---	**8.4**	4.2	9.1	7.9
204530_s_at	Thymus high mobility group box protein TOX	***TOX***	Regulation of transcription	---	**8.3**	6.6	12.5	6.0
228790_at, 221959_at	Chromosome 8 open reading frame 72	***C8orf72***	---	---	**7.6**	14.6	19.1	36.6
232136_s_at	Cortactin binding protein 2	***CTTNBP2***	---	Brain	**5.4**	7.1	4.3	6.5
233136_at	Poly(A) binding protein, cytoplasmic 5	***PABPC5***	---	Fetal brain	**5.4**	4.6	4.6	4.9
219093_at	Phosphotyrosine interaction domain containing 1	***PID1***	---	Brain (among others)	**5.1**	12.3	23.0	4.0
205773_at	Cytoplasmic polyadenylation element binding protein 3	***CPEB3***	Nucleotide binding	---	**4.5**	4.0	4.8	5.4
1560265_at	Glutamate receptor, ionotropic, kainate 2	***GRIK2***	Regulation of synaptic transmission	Cerebellum, cerebral cortex	**4.5**	3.7	5.9	7.1
238526_at	RAB3A interacting protein (rabin3)	***RAB3IP***	protein transport	Brain (among others)	**4.4**	5.9	7.2	3.2
213001_at, 219514_at	Angiopoietin-like 2	***ANGPTL2***	Development	Heart among others	**3.9**	12.2	6.0	4.0
229590_at	Ribosomal protein L13	***RPL13***	Translation	---	**3.8**	3.7	3.8	2.8
206117_at	Tropomyosin 1 (alpha)	***TPM1***	Cell motility	Muscle among others	**3.6**	4.1	4.2	3.3
202315_s_at, 217223_s_at	Breakpoint cluster region	***BCR***	Regulation of Rho protein signal transduction	---	**3.3**	4.4	7.6	4.5
234268_at	Solute carrier family 2 (facilitated glucose transporter), member 13	***SLC2A13***	Carbohydrate transport	Brain	**3.1**	3.4	4.1	3.2
228813_at, 204225_at	Histone deacetylase 4	***HDAC4***	Nervous system development	Ubiquitously expressed	**3.1**	2.6	3.9	4.4
209511_at	Polymerase (RNA) II (DNA directed) polypeptide F	***POLR2F***	Regulation of transcription	---	**3.0**	6.9	4.7	3.0
213033_s_at, 213032_at	Nuclear factor I/B	***NFIB***	Regulation of transcription, DNA-dependent, Brain development	---	**3.0**	10.7	13.8	8.2
204100_at	Thyroid hormone receptor, alpha (erythroblastic leukemia viral (v-erb-a) oncogene homolog, avian)	***THRA***	Negative regulation of transcription	Brain (among others)	**2.9**	2.9	2.2	3.7
230198_at	WD repeat domain 37	***WDR37***	---	---	**2.8**	4.0	2.8	2.6
213758_at	Cytochrome c oxidase subunit IV isoform 1	***COX4I1***	Electron transport	Ubiquitously expressed	**2.4**	2.2	2.7	3.2
221012_s_at	Tripartite motif-containing 8///tripartite motif-containing 8	***TRIM8***	---	Brain (among others)	**2.3**	3.1	3.2	3.2
202182_at	GCN5 general control of amino-acid synthesis 5-like 2 (yeast)	***GCN5L2***	Regulation of transcription, DNA-dependent	Ubiquitously expressed	**2.3**	2.5	2.4	2.3
214198_s_at	DiGeorge syndrome critical region gene 2	***DGCR2***	Cell adhesion	Brain (among others)	**2.3**	3.4	5.0	2.4
225334_at	Chromosome 10 open reading frame 32	***C10orf32***	---	---	**2.2**	3.5	4.1	3.2
210690_at	Killer cell lectin-like receptor subfamily C, member 4	***KLRC4***	Cellular defense response	Natural killer cells	**2.2**	2.2	2.0	2.2
203938_s_at	TATA box binding protein (TBP)-associated factor, RNA polymerase I, C, 110kDa	***TAF1C***	Transcription	---	**2.1**	3.2	2.6	2.1
217969_at	Chromosome 11 open reading frame2	***C11orf2***	---	---	**2.0**	2.8	3.9	3.3

39 well-characterized genes were significantly upregulated (FC > 2 and p < 0.001) in oligodendrogliomas with 1p19q codeletion in comparison to each of the 4 other sample groups. Underlined genes were studied and validated by real-time RT-PCR in the independent sample set. FD/EGFR = Fold difference of geometric means (FD) in gliomas with 1p19q codeletion in comparison to gliomas with EGFR amplification, FD/Cx = FD in gliomas with 1p19q codeletion in comparison to cortex samples, FD/CC = FD in gliomas with 1p19q codeletion in comparison to corpus callosum, FD/Stem cells = FD in gliomas with 1p19q codeletion in comparison to glioblastomas cancer stem cells [7].

### Oligodendrogliomas with 1p19q have a proneural gene expression profile

**Figure 4:**
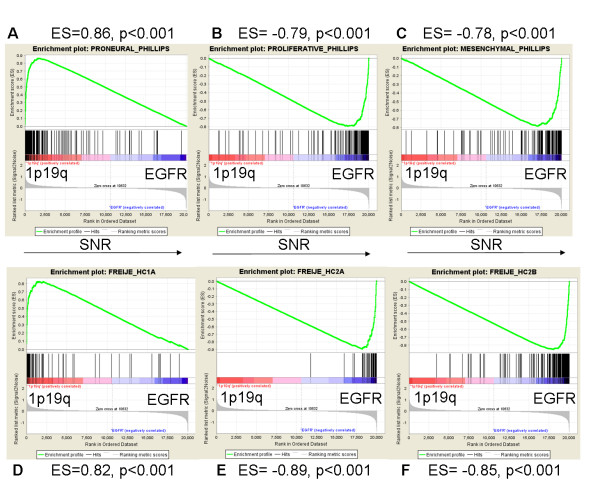
**GSEA Enrichment Score curves**. Gene set enrichment analysis (GSEA) was performed with 6 different gene sets obtained from the studies of Phillips et al. and Freije et al. Phillips' study gene sets: A: Proneural gene set (n = 220 genes), B: Proliferative gene set (n = 148 genes), C: Mesenchymal gene set (n = 126 genes). Freije's study gene sets: D: HC1A neurogenesis related gene set (n = 73), E: HC2A proliferation related gene set (n = 66 genes), F: HC2B extracellular matrix related gene set (n = 239 genes) [5, 28]. "Signal-to-Noise" ratio (SNR) statistic was used to rank the genes according to their correlation with either the 1p19q codeletion phenotype (red) or *EGFR *amplification phenotype (blue). The graph on the bottom of each panel represents the ranked, ordered, non-redundant list of genes. Genes on the far left (red) correlated the most with 1p19q codeleted samples, and genes on the far right (blue) correlated the most with *EGFR *amplified samples. On each panel, the vertical black lines indicate the position of each of the genes of the studied gene set in the ordered, non-redundant data set. The green curve corresponds to the ES (enrichment score) curve, which is the running sum of the weighted enrichment score obtained from GSEA software. A and D show that gliomas with 1p19q codeletion were significantly enriched in the proneural and neurogenesis related (HC1A) gene sets. B, C, D and E show that gliomas with *EGFR *amplification were significantly enriched in the proliferation/HC2A and mesenchymal/HC2B gene sets.

**Figure 5:**
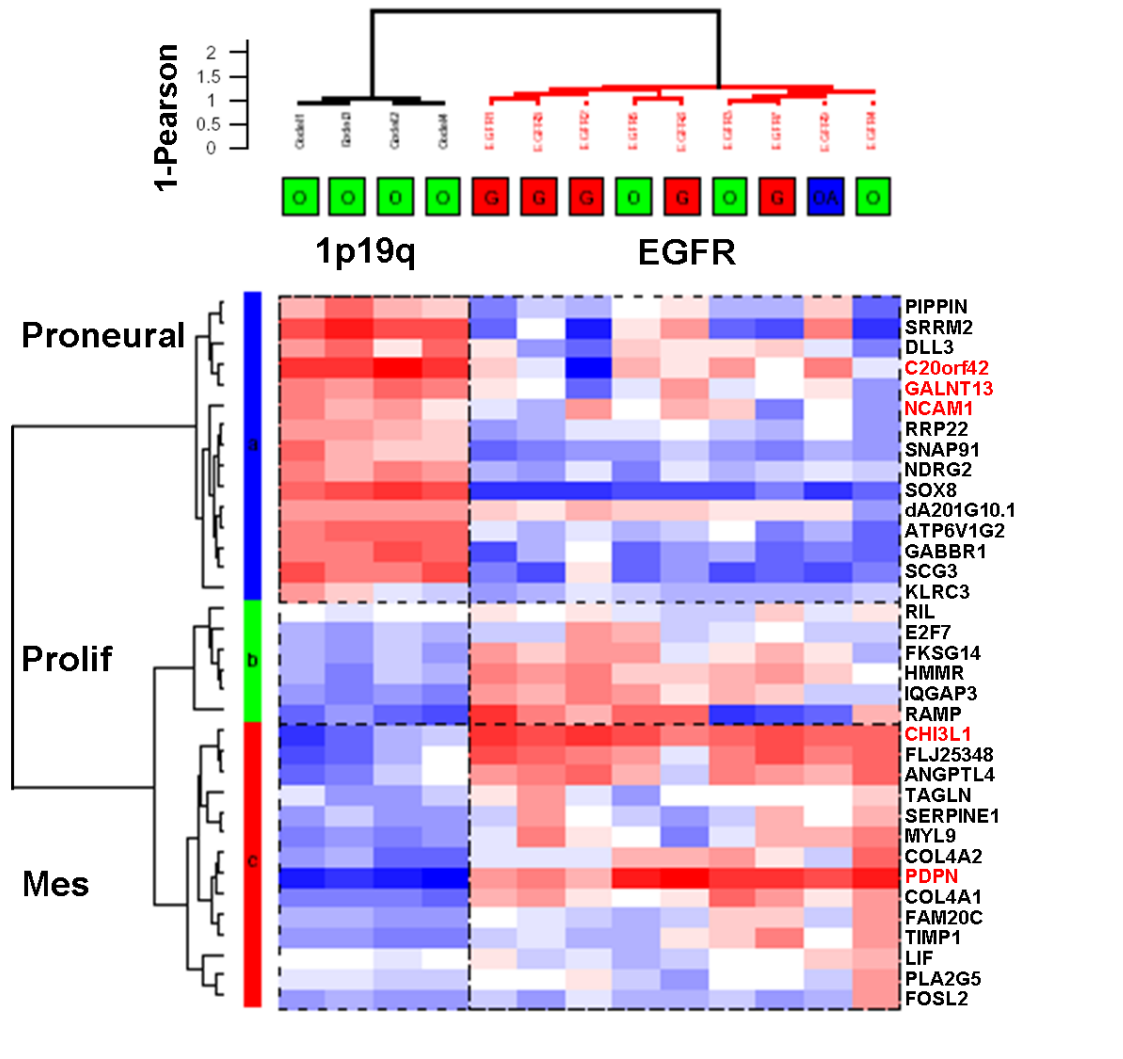
**Unsupervised clustering of the 4 gliomas with 1p19q codeletion and the 9 gliomas with *EGFR *amplification using 35 genes signature of Phillips et al. [5]**. Samples and genes were clustered using Ward's linkage and 1-Pearson correlation coefficient. 1p19q = gliomas with 1p19q codeletion, *EGFR *= gliomas with *EGFR *amplification. Gliomas with 1p19q codeletion were classified as proneural, whereas gliomas with *EGFR *amplification had both a mesenchymal and proliferative profile. In red are the genes whose expression was studied in real-time RT-PCR in an independent data set.

As gliomas with 1p19q codeletion expressed neuronal genes, we performed gene set enrichment analysis (GSEA) to study the relationship between the gene expression profile of these gliomas and the "proneural" gene expression signatures that have been described in high grade gliomas with a good prognosis (Figure 4) [5,14,28]. This test determines the over-representation of a gene set (list of genes) at the extremes (top or bottom) of the ordered, non-redundant dataset (list of all of the genes being used to compare two groups of samples). This analysis demonstrated that gliomas with 1p19q codeletion in comparison to gliomas with *EGFR *amplification were significantly enriched in the "proneural" gene set associated with good prognosis reported by Phillips et al. and in the good prognosis neurogenesis-related gene set reported by Freije et al. (HC1A gene set) [5,28]. They were also enriched in the HC1B gene set (neuronal genes) of Freije et al. In contrast, gliomas with *EGFR *amplification were enriched in gene sets associated with poor prognosis ("proliferation" and "mesenchymal" gene sets of Phillips et al., and HC2A (enriched in proliferation genes) and HC2B (enriched in extracellular matrix genes) gene sets of Freije et al.) [5,28]. Next we used the 35 genes signature developed by Phillips et al. to distinguish the three groups of high grade gliomas (proneural, proliferative and mesenchymal) in order to perform unsupervised hierarchical clustering [5]. As shown in Figure 5, the gliomas with 1p19q codeletion were classified as proneural. Thus, there was a clear association between the "proneural" gene expression profile and 1p19q codeletion.

### Real-time RT-PCR validation

**Figure 6:**
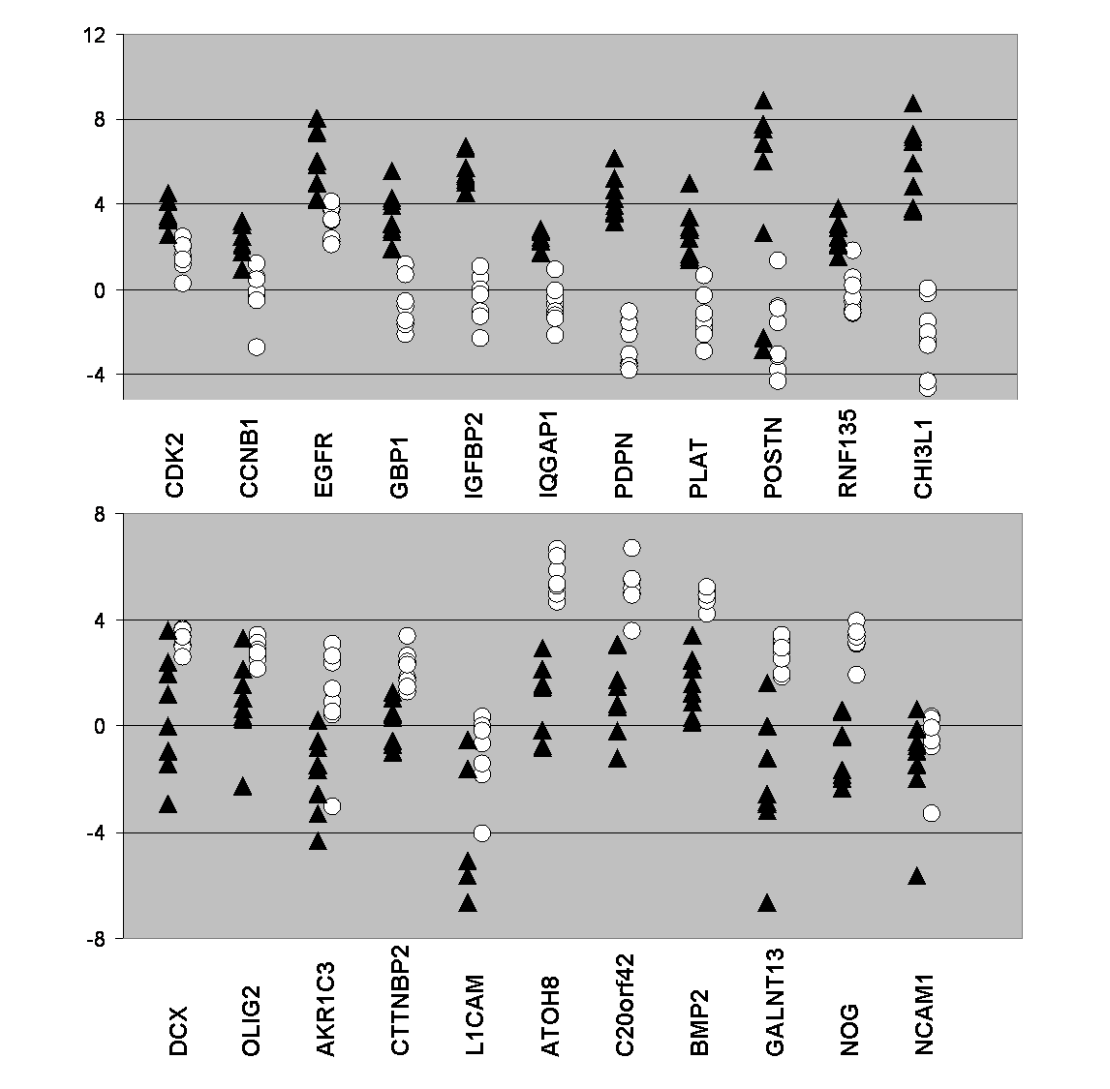
**Real-time RT PCR study of 22 genes differentially expressed between 1p19q codeleted gliomas and *EGFR *amplified gliomas**. Real-time RT-PCR study of 11 genes overexpressed in gliomas with *EGFR *amplification (top) and 11 genes overexpressed in gliomas with 1p19q codeletion (bottom) was performed in an independent data set of 16 gliomas (8 gliomas with *EGFR *amplification (triangles), 8 gliomas with 1p19q codeletion (circles)). Each dot represents the relative expression (log2 transformed) of a given gene in one glioma compared with normal brain (median expression in the 3 normal brain samples). Dots above the upper dashed line are upregulated with a fold change larger than 2 in comparison to normal brain; dots below the lower dashed line are downregulated in comparison to normal brain with a fold change larger than 2. For example, *NOG, BMP2 *and *ATOH8 *were overexpressed in all 8 gliomas with 1p19q codeletion (circles) in comparison to all 8 gliomas with *EGFR *amplification (triangles) and in comparison to normal brain. *CHI3L1, PLAT, IQGAP1, IGFFBP2 *and *GBP1*were overexpressed in all gliomas with *EGFR *amplification (triangles) in comparison to gliomas with 1p19q codeletion (circles) and in comparison to normal brain. Except for *NCAM1*, all 22 genes were differentially expressed (p < 0.05).

To validate these findings, we studied the expression of 22 selected genes differentially expressed (11 up and 11 down) between the two groups of gliomas in an independent data set of 16 gliomas (8 gliomas with *EGFR *amplification and 8 gliomas with 1p19q codeletion). This study was performed in comparison with 3 samples of normal brain obtained from epileptic surgery (Table 2, Figure 6 and see additional file 2). Using a univariate t-test, 21 out of the 22 genes studied were shown to be differentially expressed between the two groups of gliomas with a p-value < 0.05 (only *NCAM1 *was not validated). This confirmed that gliomas with 1p19q codeletion overexpressed neuronal/normal brain genes (*AKR1C3, C20orf42, CTTNBP2, L1CAM, GALNT13*) as well as genes implicated in gliogenesis and neurogenesis (*OLIG2, BMP2, NOG, DCX, ATOH8*). Except *L1CAM *and *AKR1C3*, all of these genes were also overexpressed in comparison to the normal brain samples, including two genes (*DCX, GALNT13*) known to be exclusively expressed in neuronal cells. *BMP2, NOG, C20orf42, GALNT13 *and *OLIG2 *belong to the list of proneural genes reported by Phillips [5].

**Table 2:** Real-time RT-PCR study of 22 differentially expressed genes

**Gene symbol**	**Description**	**Gene ontology**	Fold difference of geom means 1p19q/EGFR (microarray)*	Fold difference of geom means 1p19q/EGFR in validation sample set (RT-PCR)**
*AKR1C3*	Aldo-keto reductase family 1, member C3	Prostaglandin metabolism	17.5	7.1
*ATOH8*	Atonal homolog 8 (Drosophila)	Regulation of transcription	26	23.5
*BMP2*	Bone morphogenetic protein 2	Positive regulation of astrocyte differentiation	10.1	10.2
*C20ORF42*	Chromosome 20 open reading frame 42	Cell adhesion	16.2	15.7
*CTTNBP2*	Cortactin binding protein 2	---	5.4	3.9
*DCX*	Doublecortex; lissencephaly, X-linked (doublecortin)	CNS development	5.9	6.3
*GALNT13*	UDP-N-acetyl-alpha-D-galactosamine:polypeptide N-acetylgalactosaminyltransferase 13 (GalNAc-T13)	Protein amino acid O-linked glycosylation	31.7	38.4
*L1CAM*	L1 cell adhesion molecule	Nervous system development	14.6	24.5
*NCAM1*	Neural cell adhesion molecule 1	Synaptic transmission	4.8	1.7(NS)
*NOG*	Noggin	Nervous system development	11.2	18.4
*OLIG2*	Oligodendrocyte lineage transcription factor 2	Nervous system development	4.7	3.7
*CCNB1*	Cyclin B1	Mitosis	0.2	0.2
*CDK2*	Cyclin-dependent kinase 2	Mitosis	0.2	0.2
*CHI3L1*	Chitinase 3-like 1 (cartilage glycoprotein-39)	Chitin catabolism	0.01	0.003
*EGFR*	Epidermal growth factor receptor (erythroblastic leukemia viral (v-erb-b) oncogene homolog, avian)	Cell proliferation	0.1	0.2
*GBP1*	Guanylate binding protein 1, interferon-inducible, 67kDa	Immune response	0.05	0.05
*IGFBP2*	Insulin-like growth factor binding protein 2, 36 kDa	Regulation of cell growth	0.02	0.01
*IQGAP1*	IQ motif containing GTPase activating protein 1	Signal transduction	0.11	0.1
*PDPN*	Podoplanin	Positive regulation of cell motility	0.02	0.008
*PLAT*	Plasminogen activator, tissue	Proteolysis	0.07	0.06
*POSTN*	Periostin, osteoblast specific factor	Cell adhesion	0.01	0.01
*RNF135*	Ring finger protein 135	---	0.2	0.2

Fold difference of geometrical means in microarray and in real-time RT-PCR of the 22 genes studied in the independent sample set. * All genes were differentially expressed with a p-value < 0.001 except DCX (p-value = 0.004). * * All genes were differentially expressed with a p-value < 0.05 except when NS (non significant) is specified.

In the gliomas with *EGFR *amplification, we confirmed the overexpression of genes implicated in proliferation (*CCNB1, CDK2*), extracellular matrix remodeling (*PLAT, POSTN*), immune response (*GBP1*), cancer stem cell signaling (*IQGAP1*) as well as several genes known to be highly expressed in glioblastomas (*IGFBP2, CHI3L1, PDPN*). *CCNB1*, *CDK2 *belong to the proliferative gene list, and *CHI3L1 *and *PDPN*, to the mesenchymal gene list of Phillips [5].

### Alpha-internexin immunohistochemistry

**Figure 7:**
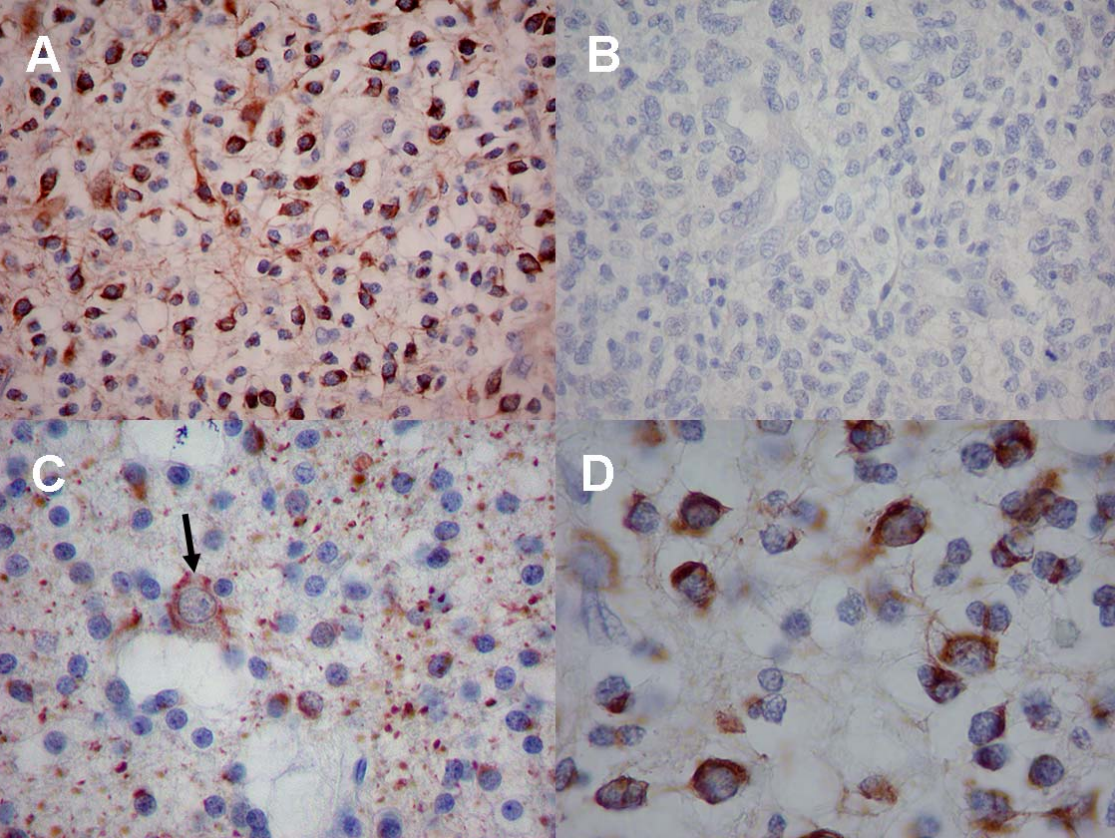
**INA immunohistochemistry in 1p19q codeleted and *EGFR *amplified gliomas**. Representative alpha-internexin (INA) immunohistostaining in oligodendrogliomas with 1p19q codeletion (A, C, D) and in glioblastomas with *EGFR *amplification (B). C: the arrow shows immunopositivity in an entrapped neuron surrounded by immunopositive tumor cells.

Finally, to validate the expression of neuronal genes in gliomas with 1p19q codeletion at the protein level, we studied the expression of the internexin neuronal intermediate filament protein alpha (INA) which was one of the neuronal genes most overexpressed in these gliomas in comparison to gliomas with *EGFR *amplification (FC = 15, p < 0.001). INA is a class-IV neuronal intermediate filament involved in the morphogenesis of neurons [29]. Immunostaining for INA was positive in all five oligodendrogliomas with 1p19q codeletion examined. Immunopositivity was observed in some normal infiltrated neurons but was mostly seen in a specific cytoplasmic perinuclear staining pattern in tumor cells (Figure 7). Between 20 to 50% of tumor cells displayed this staining, which was different from the staining observed in the infiltrated normal neurons (Figure 7). Among the five glioblastomas with *EGFR *amplification, immunostaining was negative in four and positive in a scattered pattern in one, in a region displaying some features of oligodendroglial differentiation.

## Discussion

*EGFR *amplification and whole 1p19q codeletion are mutually exclusive and predictive of completely different outcomes [3,4]. To date, no studies have compared the gene expression profile of these two types of gliomas. Indeed, among microarray studies of gliomas [5,28,30,31], only a few have compared genetically well-defined tumors [32-35]. In addition, these studies were based on LOH or FISH [32-34], and not on CGH-array. Yet, there is a need when interpreting a difference in gene expression to analyze it in relation to the genomic profile. Our data reveals clearly distinct gene expression profiles in these 2 groups of gliomas: those with *EGFR *amplification express the proliferative and mesenchymal gene set defined by Phillips et al., while 1p19q codeleted gliomas express the proneural group [5]. Moreover, gliomas with *EGFR *amplification clustered close to tumor stem cells. Indeed, the *EGFR *pathway is involved in the proliferation of normal neural stem cells and cancer stem cells [36]. This result is consistent with the fact that several studies isolated stem like tumor cells from glioblastoma but not from oligodendroglioma. Such studies did not include genetic profiles of the tumors, but data from our group suggest indeed that the capacity of cell renewal (as reflected by the formation of spheroids derived from the tumor) *in vitro *is tightly correlated with the presence of EGFR amplification (unpublished results). EGFR activation upregulates genes involved in neural stem cell proliferation: one of these genes is ASPM (abnormal spindle-like microcephaly associated) that promotes neuroblast proliferation and symmetric division and is strongly upregulated in glioblastomas. Inhibition of ASPM inhibits glioblastoma cell growth and neural stem cell proliferation [37].

A proneural/normal brain gene expression profile is a factor related to good prognosis and correlates with younger age and grade III histology, with most anaplastic oligodendrogliomas being classified as proneural [5,28]. As shown here, this gene expression profile can be determined by a simple, highly discriminating RT-PCR test, and this may be useful for clinical practice. Until now, a proneural gene expression profile has not been reported to be associated with 1p19q codeletion. In Freije's study the number of gliomas with 1p19q codeletion was too small (4 out 74 patients) to address this question [28]. In Phillips' study the genomic/transcriptomic correlation was limited to patients with astrocytoma histology, and this may have limited the possibility of finding an association between 1p19q codeletion and the proneural gene expression profile [5]. However, the authors noticed a negative correlation between the proneural gene expression profile and *EGFR *amplification, similar to the negative correlation between 1p19q codeletion and *EGFR *amplification [3,5]. Our study demonstrates that there is a strong correlation between 1p19q codeletion and the expression of proneural genes, suggesting that gliomas with a 1p19q codeletion represent a subgroup of proneural gliomas. In addition, the expression of neuronal genes in 1p19q codeleted tumors is consistent with a previous study showing selective expression of neuronal genes in oligodendrogliomas with 1p loss [33]. Whether there is a link between the good prognosis of proneural gliomas and the fact that gliomas with 1p19q codeletion display a proneural gene expression profile remains to be elucidated. We make the hypothesis that gliomas without 1p19q codeletion but with a gene expression profile similar to the 1p19q codeleted gliomas might also harbor a better prognosis.

The expression of "neuronal genes" in 1p19q codeleted gliomas can be interpreted in different ways. As advocated by some authors, this expression is probably due in part to the presence of infiltrated neurons in the tumor [30]. Indeed, 1p19q codeletion has been suggested to be more frequent in tumors with indistinct, irregular borders, which therefore, are more likely to be contaminated with normal brain tissue [38]. However, as shown here, this normal brain infiltration cannot completely explain the expression of neuronal genes by 1p19q codeleted gliomas. Indeed, these tumors only express a specific subset of neuronal genes (Figure 3). In addition, if the expression of neuronal genes was only due to infiltration of normal brain tissue, the expression pattern of the neuronal genes in these tumors would be similar to their expression in the normal brain samples, which was not the case. Furthermore, we have demonstrated that alpha-internexin (INA), a neuronal protein, was specifically expressed by 1p19q codeleted glioma tumor cells. Thus INA expression might be used as a simple surrogate marker of 1p19q codeletion. This hypothesis is currently being tested in a larger series of gliomas. Interestingly, recent ultrastructural analysis of oligodendrogliomas has shown neuronal structures such as synapses and neurosecretory granules [39]. Thus, another hypothesis for the expression of neuronal genes in 1p19q codeleted glioma tumor cells is that the cell of origin of these tumors could be a progenitor cell giving rise to both neurons and oligodendrocytes [40,41]. This progenitor has less capacity of self renewal than the more multipotent neural stem cells. This is consistent with the fact that 1p19q codeleted oligodendroglioma fails in our hands to form spheroids in vitro (unpublished data). In this setting it is interesting to note that concomitant overexpression of both BMPs and BMP antagonists, such as the concomitant overexpression of *BMP2 *and *NOG *observed in 1p19q codeleted gliomas in our study, has been demonstrated in white matter progenitor cells, which can give rise to both oligodendrocytes and neurons [42]. Another non-exclusive explanation for the expression of "neuronal" genes in oligodendrogliomas could rely on the fact that some genes involved in neurogenesis and classified as "neuronal" may also play a role in oligodendroglial development, e.g. *ASCL1/MASH1*. This proneural gene specifies a population of telencephalic oligodendrocytes [43] and is also required for oligodendrocyte development in the spinal cord [44]. On the other hand, Olig2 -implicated in oligodendroglial specification- is also involved in neurogenesis: during development, Olig2+ progenitors give rise to both motoneurons and oligodendrocytes in the ventral spinal cord, [45]. Consistently with our results, these data, illustrating the tight connection that exists between neurons and oligodendrocytes fates, bring a new light on the pathogenesis of oligodendrogliomas with 1p19q codeletion. Finally it is important to remember that current WHO classification is only based on morphological similarity between normal cells and tumor cells, and the link between oligodendrocytes and oligodendrogliomas has never been demonstrated.

## Competing interests

The authors declare that they have no competing interests.

## Authors' contributions

FD performed the major part of experiments and analysis. FD and MS drafted the manuscript. AI performed the CGH-array study and analysis. AR and SL provided the bioinformatic tools and participated to the analysis. IB and MV performed the real-time RT-PCR validation. YM, SP, JT and KM selected the samples, helped extracting the RNA and performed the immunohistochemistry validation. KHX, OD and JYD assisted with design of the study and with critical examination of the manuscript. MS conceived of and designed the study, participated in its experimental design and interpretation of results, and helped edit the manuscript. All authors read and approved the final manuscript.

## Supplementary Material

Additional file 1 Complete list of genes differentially expressed (t-test, p < 0.001) between 1p19q codeleted oligodendrogliomas (n = 4) and 1) gliomas with EGFR amplification (n = 9), 2) cerebral cortex samples (n = 5), 3) corpus callosum samples (n = 5) 4) Beier's et al. gliomas cancer stem cells (n = 6) [7]. Click here for file

Additional file 2 Detailed results of the 22 genes studied by real-time RT-PCR in the independent sample set of 8 oligodendrogliomas with 1p19q codeletion and 8 gliomas with EGFR amplification. Click here for file
